# Unveiling the repressive mechanism of a PPS-like regulator (PspR) in polyhydroxyalkanoates biosynthesis network

**DOI:** 10.1007/s00253-024-13100-x

**Published:** 2024-03-18

**Authors:** Junyu Chen, Yinglu Cui, Shengjie Zhang, Bian Wu, Jing Han, Hua Xiang

**Affiliations:** 1 State Key Laboratory of Microbial Resources, Institute of Microbiology, Chinese Academy of Sciences, Beijing, 100101 People’s Republic of China; 2 College of Life Science, University of Chinese Academy of Sciences, 100049 Beijing, People’s Republic of China

**Keywords:** Haloarchaea, Poly(3-hydroxybutyrate-*co*-3-hydroxyvalerate) biosynthesis, PPS-like regulator (PspR), Repressor, Functional conversion

## Abstract

**Abstract:**

Poly(3-hydroxybutyrate-*co*-3-hydroxyvalerate) (PHBV) is a type of polyhydroxyalkanoates (PHA) that exhibits numerous outstanding properties and is naturally synthesized and elaborately regulated in various microorganisms. However, the regulatory mechanism involving the specific regulator PhaR in *Haloferax mediterranei*, a major PHBV production model among Haloarchaea, is not well understood. In our previous study, we showed that deletion of the phosphoenolpyruvate (PEP) synthetase-like (*pps*-like) gene activates the cryptic *phaC* genes in *H. mediterranei*, resulting in enhanced PHBV accumulation. In this study, we demonstrated the specific function of the PPS-like protein as a negative regulator of *phaR* gene expression and PHBV synthesis. Chromatin immunoprecipitation (ChIP), in situ fluorescence reporting system, and in vitro electrophoretic mobility shift assay (EMSA) showed that the PPS-like protein can bind to the promoter region of *phaRP*. Computational modeling revealed a high structural similarity between the rifampin phosphotransferase (RPH) protein and the PPS-like protein, which has a conserved ATP-binding domain, a His domain, and a predicted DNA-binding domain. Key residues within this unique DNA-binding domain were subsequently validated through point mutation and functional evaluations. Based on these findings, we concluded that PPS-like protein, which we now renamed as PspR, has evolved into a repressor capable of regulating the key regulator PhaR, and thereby modulating PHBV synthesis. This regulatory network (PspR-PhaR) for PHA biosynthesis is likely widespread among haloarchaea, providing a novel approach to manipulate haloarchaea as a production platform for high-yielding PHA.

**Key points:**

*• The repressive mechanism of a novel inhibitor PspR in the PHBV biosynthesis was demonstrated*

*• PspR is widespread among the PHA accumulating haloarchaea*

*• It is the first report of functional conversion from an enzyme to a trans-acting regulator in haloarchaea*

**Supplementary Information:**

The online version contains supplementary material available at 10.1007/s00253-024-13100-x.

## Introduction

Polyhydroxyalkanoates (PHA) have emerged as highly promising polymers that accumulate in certain archaea and bacteria (Reddy et al. 2003; Chen and Wu 2005). These polymers are naturally synthesized by these microorganisms under unbalanced nutrition conditions with an excess of carbon sources (Reddy et al. 2003). Due to their biodegradability, biocompatibility, as well as advantageous thermoplastic and mechanical properties with great optimization potential, PHA is considered a promising alternative to petroleum-based plastics (El-Malek and Steinbuchel 2021). Among the various types of PHA, poly(3-hydroxybutyrate-*co*-3-hydroxyvalerate) (PHBV) stands out due to incorporation of 3-hydroxyvalerate (3HV) monomers into the PHB chain. This enhances the flexibility and mechanical properties of the polymer (Han et al. 2015). Extensive research has demonstrated the potential of PHBV with varying 3HV mol% as a biomaterial for tissue engineering and wound healing (Han et al. 2017; Xue et al. 2018; Kim et al. 2020).

For the biosynthesis of PHA, the key enzymes participating in the supply and polymerization pathways of PHA monomer have already been extensively studied in halophiles (Lu et al. 2008; Han et al. 2013; Fu et al. 2014). Among them, *β*-ketothiolase (PhaA), *β*-ketoacyl-CoA reductase (PhaB), and PHA synthase (PhaC) are the most well-known enzymes directly involved in PHA synthesis. Unlike most PHA-accumulating halophilic bacteria utilizing the single-subunit PhaC, a Class I type PHA synthase, to biosynthesize PHB, most halophilic archaea utilize the two-subunit PhaEC, a Class III type PHA synthase, to biosynthesize PHBV (Lu et al. 2008). Additionally, the PHA-related gene cluster is mostly conserved and well arranged in haloarchaea, such as in the order of *phaJ-phaR-phaP-phaE-phaC* in *H. mediterranei* (Liu et al. 2016).

The regulation of PHA synthesis is complex and is influenced by various factors, including environmental nutrients, key enzymes, and regulatory proteins (Sagong et al. 2018). Many attempts have been made to unravel the regulatory networks of PHA synthesis in several model species of bacteria and haloarchaea, such as *Cupriavidus necator* and *H. mediterranei* (Kessler and Witholt 2001; Velázquez-Sánchez et al. 2020; Mitra et al. 2021). PHA synthesis in both *C. necator* and *H. mediterranei* is controlled by at least a negative regulatory factor, PhaR, which is a DNA-binding protein. In *C. necator*, PHB accumulation is also regulated by PhaM and the PTS system (Pötter et al. 2002, 2005; Kaddor and Steinbüchel 2011; Wahl et al. 2012; Pfeiffer and Jendrossek 2014; Cai et al. 2015).

In *H. mediterranei*, PhaP is the predominant structure protein (phasin) on the PHBV granules involved in PHBV accumulation, while PhaR acts as a negative regulator of the *phaRP* operon (Cai et al. 2012, 2015). In our previous research, a novel protein named PPS-like (renamed as PspR throughout this manuscript) that showed high homology but lacked the same enzyme activity as phosphoenolpyruvate synthetase (PPS) was found to be a negative regulator of a cryptic *phaC*1 expression (Chen et al. 2020). Deletion of this *pps*-like gene (renamed as *pspR* gene throughout this manuscript) relieved the negative effect from *phaC*1 expression and thereby restored PHBV accumulating ability in Δ*phaC* mutant (Chen et al. 2020). Interestingly, RNA-seq data revealed the upregulation of some other genes involved in the PHBV monomer supplying (*bktB*, *phaB1*, *phaB2*, and *phaJ*) and PHBV biosynthesis (*phaR*, *phaP*, *phaE*, and *phaC*) in ΔEPSΔ*pspR *(Chen et al. 2019). However, further verification is required to determine the specific characteristics of this PspR repressor in *H. mediterranei*.

It is worth noting that *H. mediterranei* is capable of accumulating PHBV using multiple 3HV-unrelated cheap carbon sources (Hou et al. 2015). Various types of kitchen wastes such as chitins from shrimp shells, as well as industrial wastes such as olive mill wastewater, vinasse, cheese whey, and rice-based ethanol stillage, have been efficiently utilized by *H. mediterranei* as low-cost substrates for PHBV production (Bhattacharyya et al. 2012, 2015; Hou et al. 2014; Pais et al. 2016; Alsafadi and Al-Mashaqbeh 2017). Combined with other unique features such as resistance to contamination, absence of reported phages, and easy PHBV extraction, *H. mediterranei* demonstrates great promise as a cell factory for PHBV synthesis (Zhao et al. 2013; Shih et al. 2015; Mitra et al. 2020). However, as a promising PHBV factory, *H. mediterranei* remains to be thoroughly investigated to understand the regulation of PHBV synthesis and to gain more available strategies for industrial improvement. In this study, we aim to reveal the mechanisms of the candidate repressor PspR in PHBV biosynthesis and develop novel strategies to enhance PHBV production.

## Materials and methods

### Strains, medium, and culture conditions

The strains used in this study are listed in Table S1. *Escherichia coli* DH5α and *E. coli* JM 110 were used to construct plasmids and eliminate methylation of plasmids, respectively. They were cultivated in a Luria–Bertani medium (Sambrook et al. 1989) at 37 °C. Ampicillin was added at a concentration of 100 μg/mL when required. *H. mediterranei* ΔEPS, a uracil-auxotrophic strain with knock-out of the *pyrF* gene and exopolysaccharides (EPS) related genes (Liu et al. 2011), and its gene deletion mutants were grown in AS-168 medium (Han et al. 2013) at 37 °C and were used as seed culture. The seed culture was then inoculated in a 250-mL shake flask in an MG medium (PHA production medium with glucose as the sole carbon source) (Chen et al. 2019) with 10 g/L glucose as a carbon source. Uracil was added at a concentration of 50 μg/mL for the cultivation of *pyrF*-deleted strains. *Haloferax volcanii* H1424 was used for haloarchaea protein expression and was cultured in Hv-YPC medium (Liu et al. 2015) at 45 °C supplemented with 50 μg/mL uracil and 20 μg/mL thymidine (Chen et al. 2020).

### Construction of plasmids and mutant strains

The plasmids and primers used for green fluorescent protein (GFP) expression, and protein point mutations in this study are summarized in Table S1 and Table S2. The plasmid for overexpression of PspR in *H. mediterranei* strains (Table S1) was constructed based on the shuttle plasmid pWL502. Plasmids for the replacement of PHA-related genes with soluble-modified, red-shifted green fluorescent protein (smRSGFP) genes were constructed based on the suicide plasmid pHFX. The plasmids for protein expression in *H. volcanii* H1424 were constructed based on the plasmid pTA06. The transformation of *H. mediterranei* and *H. volcanii* was performed by the PEG-mediated method (Cline et al. 1989). Gene mutant construction was performed by pop-in/pop-out method, and verification was carried out using the PCR method, as described previously (Liu et al. 2011).

### Chromatin immunoprecipitation and quantitative real-time PCR (ChIP-qPCR)

A C-terminal tagged PspR-Myc protein was expressed in a *pspR gene* knockout mutant for ChIP analysis. The cells at the late-exponential-phase (36 h) were cultured in YE^−^ medium and collected for ChIP assay according to Wilbanks et al. (2012) and Cai et al. (2015). Protein A-Sepharose CL-4B beads (GE Healthcare) and DynaMag™-Magnet Spin (Thermofisher, USA) were used for protein binding and purification, respectively. The qPCR (quantitative PCR) was performed and analyzed by ViiATM 7 Real-Time PCR System (Applied Biosystems, USA) with Kapa SYBR Fast qPCR master mix (KM4101, Kapa Biosystems). Primers used for qPCR to detect the amount of the targeted DNA fragments are listed in Table S2. The amount of DNA fragments was calculated according to a quantitative standard curve constructed by relating the log of the initial number of templates in each standard to a fractional cycle number derived from each curve (Pryor and Wittwer 2006). We named the total amount of DNA serving as the input sample and set the mock sample treated without anti-Myc as the inner control. The relative abundance of each region in both the enriched DNA samples by ChIP (output) and the total DNA samples (input) was calculated by normalization to the abundance of the mock sample as follows: ΔCt_output_ = Ct_output_-Ct_input_, ΔCt_mock_ = Ct_mock_-Ct_input_, and ΔΔCt_output_ = ΔCt_output_-ΔCt_mock_. The final fold enrichment was calculated using the formula 2^(ΔΔCtoutput)^. Samples were detected in triplicate from three independent experiments, and representative results from one biological replicate are presented.

### Protein expression, purification, and electrophoretic mobility shift assay (EMSA)

PspR and its mutants were expressed in *H*. *volcanii* H1424, while RPH_*Lm*_ was expressed in *E. coli* BL21 (DE3) (Novagen, China). The proteins were purified using a Ni column (GE, USA) and HiLoad Superdex 200 prep grade (GE, USA) as previously described (Chen et al. 2019). The double-stranded DNA fragment (211 bp) of *phaR* promoter was amplified by PCR using the primer pair of phaR-Pro-F/phaR-Pro-R (Table S2). The purified PCR product as the probe for EMSA was labeled with biotin by using a Chemiluminescent Biotin-labeled Nucleic Acid Detection Kit (Beyotime, China). The EMSA reaction was performed using a Chemiluminescent EMSA Kit (Beyotime, China) with minor modifications as previously described (Chen et al. 2020).

### Relative fluorescence units (RFU) detection for promoter activity assay

Strains harboring the GFP reporter were cultured in an MG medium. A total of 200 μL cells at the log phase (24 h) and stationary phase (48 h) were transferred into the 96-well plate to measure the optical density at 600 nm (OD_600_) and the fluorescence intensity (excitation, 488 nm; emission, 509 nm) using a Synergy H4 hybrid microplate reader (BioTek Instruments, USA), with MG medium serving as the blank control. The fluorescence intensity was normalized against the cell density, which is calculated as RFU = fluorescence intensity / OD_600_.

### Computational Modeling

The PspR model was built using the Rosetta ab initio and comparative modeling methodology. The initial step involves screening the query sequence for regions that possess a homologue with an experimentally characterized structure with BLAST, PSI-BLAST, FFAS03, and 3D-Jury. The sequence was cut into putative domains based on matches to known families and structures, multiple sequence information, and predicted secondary structure information. The fragment files were generated using the Robetta online server (http://robetta.bakerlab.org/). Then, detected parents and the regions of the query were assigned to the template-based modeling protocol with multiple sequence alignment (MSA) based methods. Once the chain is completely assembled, the sidechains of the models are repacked using a Monte-Carlo algorithm. After refinement, the top-scoring model among the obtained models was selected for further analysis.

### Phosphate transferase activity detection

The 200 μL reaction mixture contained 1 mM Rifampin (RIF), 2 mM ATP, 5 mM MgCl_2_, 2 M NaCl, 50 mM Tris·HCl (pH 8.0), 2 M KCl, and 6 μg protein. HPLC was used to detect the substrate (RIF) and product (RIF-P) during the reaction at 0, 10, 30, and 60 min time points. The retention time of RIF is about 11 min, and the retention time of RIF-P (phosphorylated rifampin) is about 12 min.

### Sequence analysis and database

Query sequences were accessed from the National Center for Biotechnology Information (NCBI) Protein Database. Sequence blast was analyzed via the Protein–protein Blast server of NCBI.

### Statistical analysis

The results were presented as the mean ± standard error of three independent replicates. Significant differences among groups were identified by one-way analysis of variance (ANOVA), with statistical significance of “*”, “**”, and “***” defined at* P* value < 0.05, 0.01, and 0.001 separately.

## Results

### ChIP-qPCR reveals the binding of PspR to P_*phaR*_ and P_*phaC1*_ in vivo

**Fig. 1:**
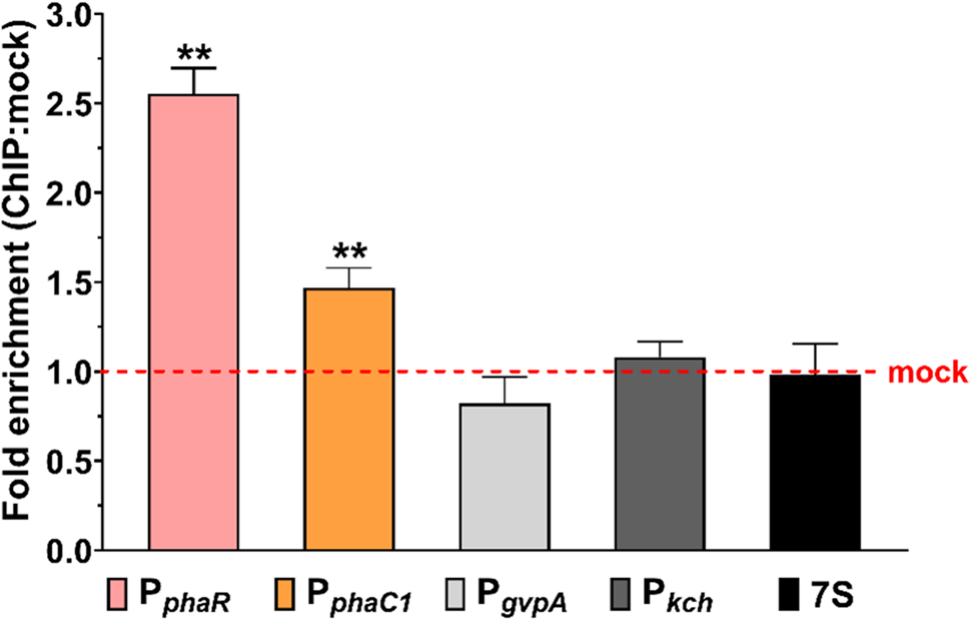
DNA enrichment assay of the PspR-Myc fusion protein. P_*phaR*_ (pink), P_*phaC1*_ (orange), P_*gvpA*_ (light grey), and P_*kch*_ (dark grey) mean that the detected promoter regions and 7S (dark) represent the distal DNA region control for this experiment. Mock sample, standing for the DNA fragments non-special binding to PspR is used for normalization. *gvpA*: encoding gas vesicle structural protein. *kch*: encoding ion channel protein. Raw data is presented in Table S3. Error bars show standard deviations (*n* = 3). Statistical significance is defined as **P < 0.01

According to our previous work, PspR might be a negative regulator of the *phaC1* gene by binding to its promoter region (Chen et al. 2020). To demonstrate the DNA-binding ability of PspR in vivo, a ChIP-qPCR assay was performed. The vector for expression of the PspR-Myc-tag fusion, pWL502-PspR-Myc, was constructed based on the plasmid pWL502. This expression vector was transformed into ΔEPSΔ*pspR* strain to obtain the ΔEPSΔ*pspR* (PspR-Myc) strain. Then, we cultured ΔEPSΔ*pspR* (PspR-Myc) strain in YE^−^ (yeast extract free medium derived from AS-168 medium). Cells in the late log phase were collected for ChIP assay. We used qPCR for DNA-binding detection as described in the method. Probes of four genes promoter regions, *phaR*, *phaC1*, *gvpA,* and *kch* gene, including BRE and TATA box, were designed for DNA amount detection (signed as P_*phaR*_, P_*phaC1*_, P_*gvpA*_, and P_*kch*_ respectively), along with housekeeper gene 7S as a distal control. Among them, *phaR*, *phaC1*, and *gvpA* genes were upregulated after *pspR* knock-out, and *kch* was not significantly regulated (Chen et al. 2019). As shown in Fig. 1, the fragments P_*phaR*_ (2.55-fold) and P_*phaC1*_ (1.47-fold) were remarkably enriched by PspR with Myc tagged, while no significant enrichment of P_*gvpA*_ (0.82-fold), P_*kch*_ (0.98-fold) and the distal control 7S (0.99-fold) were observed (raw data in Table S3). Results indicated that PspR had a specific binding capacity to the promoter regions of *phaR* and *phaC1* in vivo, the latter of which corresponded with the previous conclusion (Chen et al. 2020).

### EMSA indicates the specific binding activity of PspR to P_*phaR*_ in vitro

**Fig. 2:**
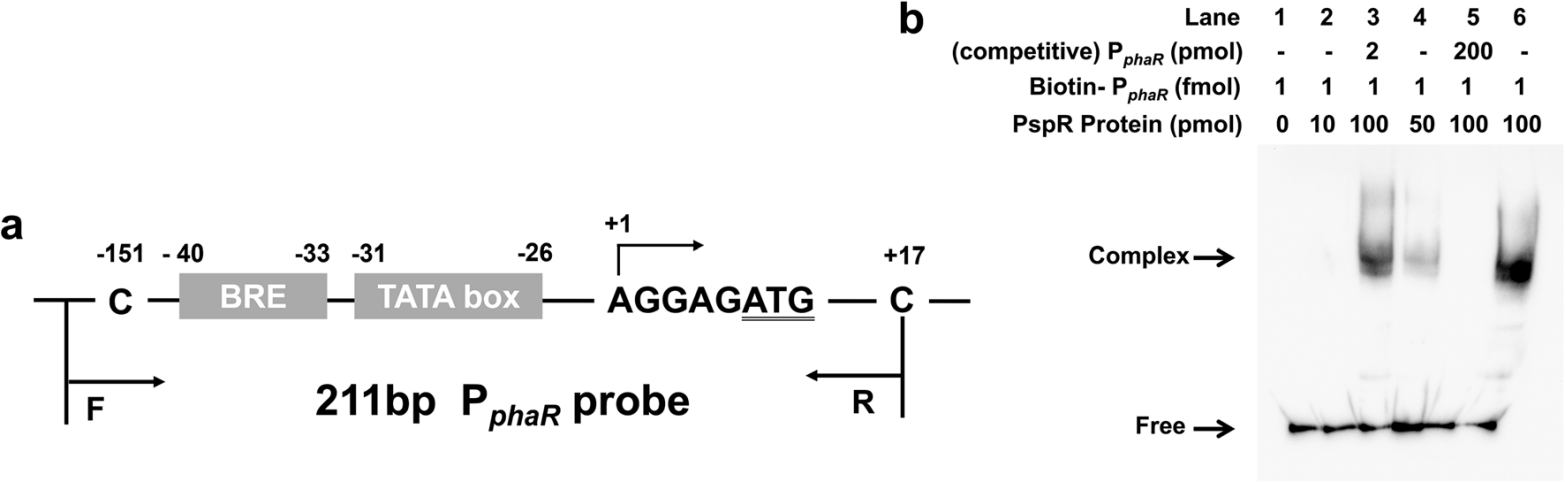
Binding specificity of PspR to P_*phaR*_ by EMSA. **a** The P_*phaR*_ nucleic acid fragments are obtained by PCR with primer pairs of phaR-Pro-F/phaR-Pro-R. **b** The purified nucleic acids are labeled with biotin by labeling kit, and the quantity of probes in each reaction is adjusted to 1 fmol, along with gradient quantities of PspR protein were set as 0 pmol (lane 1), 10 pmol (lane 2), 50 pmol (lane 4), and 100 pmol (lane 3, 5, and 6), respectively. Complex: the shifted protein-DNA complexes. Free: the free probe substrates. Competitive P_*phaR*_ is the P_*phaR*_ nucleic acid fragment without labeling biotin acting as a rivalrous probe (lane 4)

According to the result of ChIP-qPCR in vivo, the PspR could bind to the P_*phaR*_ region. Hence, we detected the specific binding activity of PspR to P_*phaR*_ by EMSA assay in vitro. We designed a biotin-labeled probe of P_*phaR*_ (described above), named Biotin-P_*phaR*_ (Fig. 2a). EMSA was performed as described previously. Shifted protein–DNA complex bands could be observed as the molar ratio of protein: nucleotide probe reached 50000:1 (Fig. 2b, lane 4). As the protein concentration increased continuously (100000:1, lane 6), the intensity of the blocking band increased as well. Meanwhile, competitive probes (P_*phaR*_ probes without biotin labeling, lanes 3 and 5) were added, and a decreased binding efficiency was observed in lane 3. When the competitive probe/biotin probe ratio reached 200000:1, there was no shifted complex band in lane 5. Taken together, it might be proposed that PspR showed a specific and weak binding ability to the P_*phaR*_ promoter region to suppress the transcription initiation of *phaRP*.

### PspR inhibits the P_*phaR*_-promoting activity

**Fig. 3:**
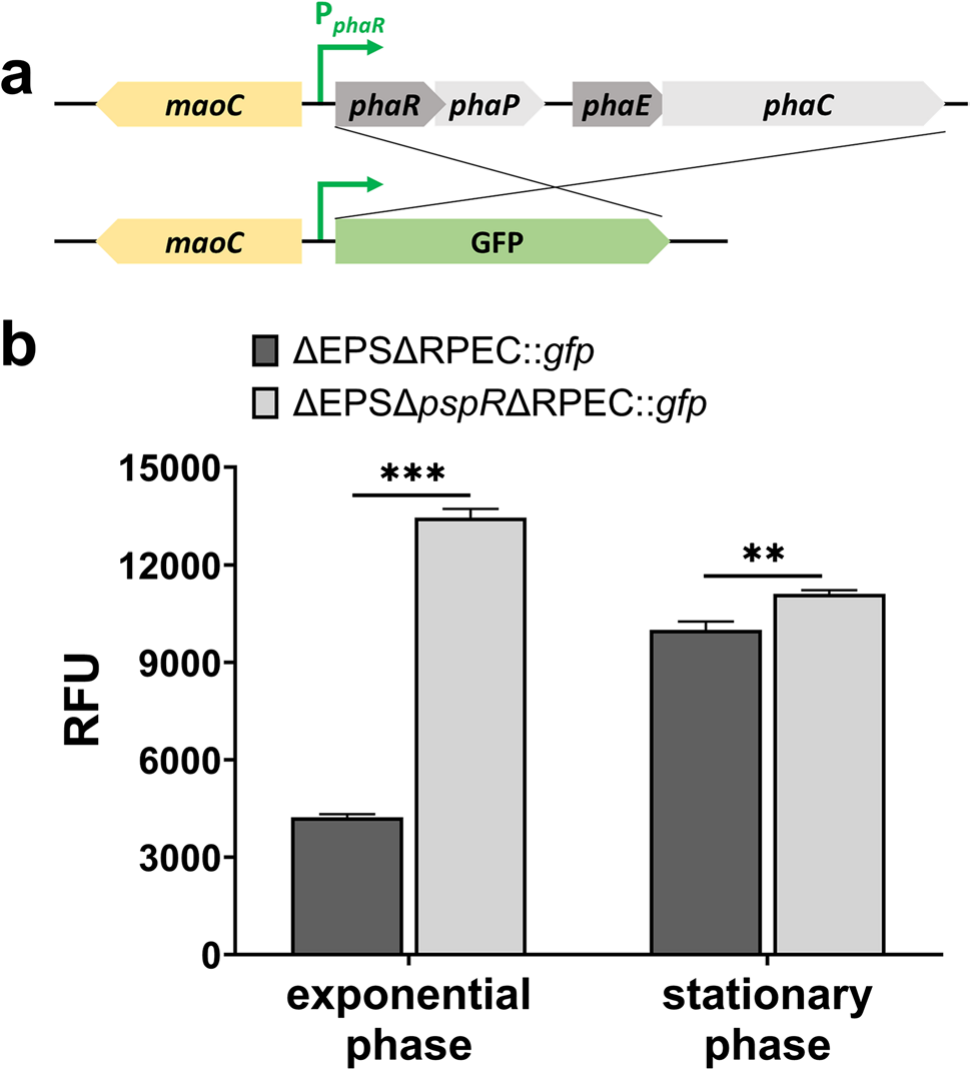
GFP expression is upregulated in ΔEPSΔ*pspR*ΔRPEC::*gfp* strain.** a** Structure of GFP report system; **b** GFP expression of ΔEPSΔRPEC::*gfp* (dark grey) and ΔEPSΔ*pspR*ΔRPEC::*gfp* (light grey) in exponential and stationary phase. RFU represented the relative fluorescence units expressed, which is calculated as RFU = fluorescence intensity (excitation wavelength 488 nm, emission wavelength 509 nm) / OD_600_. Raw data are presented in Table S4. Error bars show standard deviations (*n* = 3). Statistical significance is defined as ***P < 0.001, and **p < 0.01

ChIP-qPCR results showed the strongest binding activity of PspR to P_*phaR*_ among the detected promoter regions, and this phenomenon was supported by the EMSA result. Furthermore, we constructed a P_*phaR*_-initiated GFP fluorescence reporting system on chromosome to detect the functional effect of PspR on P_*phaR*_-promoting activity. We chose smRSGFP (soluble-modified, red-shifted green fluorescent protein, shortly named GFP), which is active in haloarchaea, as a reporter protein (Reuter and Maupin-Furlow 2004). As shown in Fig. 3a, we constructed the strain ΔEPSΔRPEC::*gfp* and ΔEPSΔ*pspR*ΔRPEC::*gfp*, in which the ORF (open reading frame) of the PHA-related genes cluster (open reading frame of *phaR*-*phaP*-*phaE*-*phaC* region) was replaced by GFP-ORF in situ, retaining P_*phaR*_ for the transcription of *gfp* gene. Then, we cultured these two strains in MG medium and performed fluorescence intensity detection of GFP and OD_600_ with a microplate reader at 24 h and 48 h. RFU (relative fluorescence units) of the two strains differed both in the log phase and stationary phase, which increased by 3.18-fold and 1.11-fold, respectively, after *pspR* knockout (Fig. 3b, raw data in Table S4). This data suggested that PspR indeed acted as an inhibitor of P_*phaR*_ promoter, which mainly occurred in the log phase. In conclusion, these results revealed that the absence of PspR could enhance the promoter activity of P_*phaR*_, demonstrating that PspR is a transcriptional repressor of both *phaRP* and *phaC1* by binding to their promoter regions.

### Structural modeling of DNA-binding domain in PspR

**Fig. 4:**
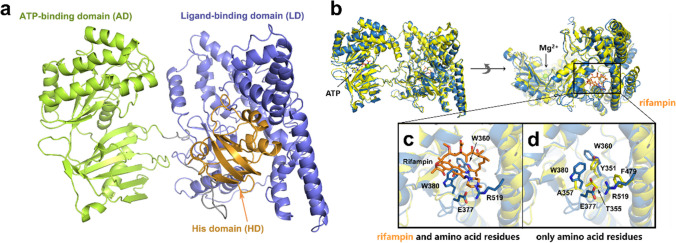
Predicted structure of PspR. **a** The AD, LD, and HD of PspR are colored green, purple, and gold, respectively. **b** Structure superimposition of PspR model with RPH-rifampin-ATP bound complex. The PspR model and RPH-rifampin complex are colored yellow and blue, respectively. ATP and rifampin are shown as sticks and rifampin is orange; Mg^2+^ is shown as a green sphere. **c** The steric clash between rifampin (orange) and amino acid residues (W360 and W380, shown as blue) in the superimposed structures. Both rifampin and residues are shown. **d** Candidate key residues in LD region of PspR model and RPH-rifampin bound complex. Only amino acid residues are shown. *W* tryptophan, *R* arginine, *E* glutamic acid, *A* alanine, *T* tryptophan, *Y* tyrosine, *F* phenylalanine

According to multiple sequence alignment analysis in the UniProt database, the amino acid sequence of PspR showed a low similarity with all reported members of the PEP utilization family (less than 40% sequence identity). Interestingly, PspR exhibited a certain degree of sequence identity (35%) with rifampin phosphotransferase (RPH_*Lm*_, derived from *Listeria monocytogenes*, PDB ID: 5HV1). Traditional template-based homology modeling could not provide a satisfactory prediction of the three-dimensional structure of PspR. Hence, we performed de novo structure prediction, which includes both Rosetta ab initio and comparative modeling methodology to build the PspR mode. According to the predicted model, the overall PspR structure presented a conserved saddle-like shape of phosphotransferase consisting of three domains: the ATP-binding domain (AD), the ligand-binding domain (LD), and the His domain (HD), which contained a conserved His residue essential for phosphate transfer (Fig. 4a). The AD and LD form two flaps of the saddle, whereas the C-terminal HD binds to the RD from the concave side of the “saddle.” Through a flexible loop, the HD might swing between the AD and RD to transfer a phosphate from ATP to the ligand. However, huge structural differences in the LD region in PspR model were observed through a structure superimposition of PspR model with an RPH-rifampin bound complex (PDB ID: 5HV1) (Fig. 4b, Fig. S1). DNA-binding region predicted by the DP-bind online server (http://lcg.rit.albany.edu/dp-bind/) implied that the LD region might be responsible for the binding of DNA. As shown in Fig. 4c and d, the steric clash between rifampin and two aromatic residues (W360 and W380, W: tryptophan) was observed, which might explain the rearrangement of the LD active site. Apart from this, glutamic acid residue (E377) and arginine residue (R519), which could form salt-bridge interactions at the surface of the LD region, might have an impact on the architecture of the ligand-binding domain and act as “gatekeepers” to block the entrance and exit of the substrate. These domains implied the DNA-binding capacity of PspR.

### Crucial sites of DNA-binding domain in PspR

**Fig. 5:**
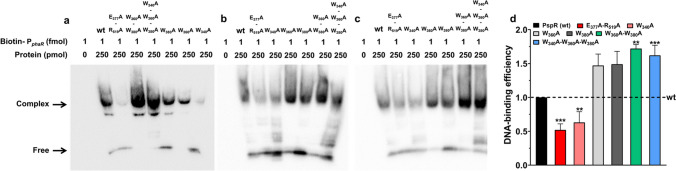
Different binding specificity of mutated PspRs (mPspRs) to P_*phaR*_ region determined by EMSA. **a, b, c** Independent repeated experiment results. The complex stands for the shifted protein-DNA complex, and free stands for the free probe substrate. **d** Statistics of the DNA-binding efficiency of mPspRs are presented in (**a**), (**b**), and (**c**). PspR and mPspRs are colored in dark (PspR, wt), red (E_377_A-R_519_A), pink (F_340_A), grey (W_360_A), dark grey (W_380_A), green (W_360_A-W_380_A), and blue (F_340_A-W_360_A-W_380_A), respectively. Raw data are presented in Table S5. Error bars show standard deviations (*n* = 3). Statistical significance is defined as **P < 0.01 and ***P < 0.001

As described in Fig. 4, there are four candidate crucial sites for the PspR LD domain, two aromatic residues (W360 and W380), glutamic acid residue (E377), and arginine residue (R519). In addition, phenylalanine residue (F340) in the hydrophobic pocket may also play an important role in DNA binding or maintaining structural stability (Fig. S1). To explore the role of these key residues that may participate in DNA binding, we constructed six kinds of single or grouped position mutation proteins (mPspR), named as F_340_A, W_360_A, W_380_A, E_377_A-R_519_A, W_360_A-W_380_A, and F_340_A-W_360_A-W_380_A. EMSA was conducted with the molar ratio of protein: nucleotide probe reaching 250, 000:1 (Fig. 5a, b, and c), along with the original PspR as the contrast. EMSA result presented different binding capabilities of mutant proteins to P_*phaR*_ probe. Then, we calculated the relative quantity of binding efficiency by ImageJ software. As shown in Fig. 5d (raw data in Table S5), the E_377_A-R_519_A mutant resulted in a decrease by half of DNA binding capability, inferring that electrostatics interactions at the surface of the LD region are necessary for DNA binding. In contrast to the decreased DNA binding efficiency in the E_377_A-R_519_A mutant, DNA binding activities of W_360_A, W_380_A, and W_360_AW_380_A increased by approximately 1.47, 1.49, and 1.72-fold, respectively. Based on these results, we inferred that the steric clash between the LD region and ligand constructed by W_360_ and W_380_ was eliminated after mutation, thus enhancing the binding efficiency. In addition, the single mutation of F_340_ caused a 27% decrease in DNA binding efficiency, while the grouped mutation of F_340_, W_360_ with W_380_ still caused a 1.62-fold increase in DNA binding efficiency. Combining these results, we hypothesized that the significance of F_340_ in maintaining the stability of the hydrophobic pocket in the LD region is reduced when the steric clash is eliminated.

**Fig. 6:**
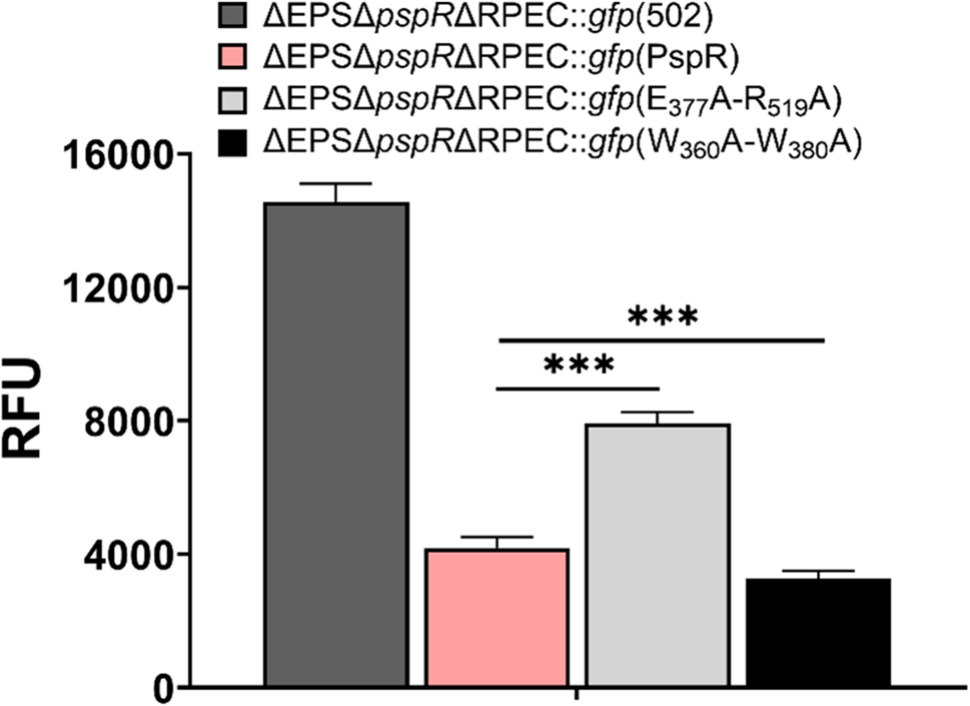
Different binding specificity of mPspRs to P_*phaR*_ region determined by fluorescent reporter system. GFP expression of ΔEPSΔ*pspR*ΔRPEC::*gfp*(502), ΔEPSΔ*pspR*ΔRPEC::*gfp*(PspR), ΔEPSΔ*pspR*ΔRPEC::*gfp*(E_377_A-R_519_A), and ΔEPSΔ*pspR*ΔRPEC::*gfp*(E_377_A-R_519_A) in exponential phase are detected. RFU represents the relative fluorescence units expressed, which is calculated as RFU = fluorescence intensity (excitation wavelength 488 nm, emission wavelength 509 nm) / OD_600_. Raw data are presented in Table S6. Error bars show standard deviations (*n* = 3). Statistical significance is defined as ***P < 0.001

In addition, we compared the binding efficiency of mPspRs (E_377_A-R_519_A and W_360_A-W_380_A) to P_*phaR*_ in vivo (Fig. 6). Based on the fluorescent reporter system in Fig. 3a, we expressed PspR, E_377_A-R_519_A, and W_360_A-W_380_A in ΔEPSΔ*pspR*ΔRPEC::*gfp* strain respectively, which were named as ΔEPSΔ*pspR*ΔRPEC::*gfp*(PspR), ΔEPSΔ*pspR*ΔRPEC::*gfp*(E_377_A-R_519_A), and ΔEPSΔ*pspR*ΔRPEC::*gfp*(E_377_A-R_519_A). As shown in Fig. 6 (raw data in Table S6), the RFU of ΔEPSΔ*pspR*ΔRPEC::*gfp*(E_377_A-R_519_A) strain increased by 1.89-fold, which indicated a decrease of binding efficiency of mPspR(E_377_A-R_519_A) to P_*phaR*_. Meanwhile, the RFU of ΔEPSΔ*pspR*ΔRPEC::*gfp*(W_360_A-W_380_A) strain decreased by 22%, which indicated an increase in binding efficiency of mPspR (W_360_A-W_380_A) to P_*phaR*_. Taken together, these findings clearly implicated the crucial role of the LD region of PspR in DNA binding and highlighted that the regulatory activity of PspR can be modulated by altering the key amino acids within the LD region.

## Discussion

*H. mediterranei* is a promising industrial strain for PHBV production, as it possesses a peculiar set of enzymes and pathways dedicated to PHBV synthesis and regulation (Chen et al. 2020; Lu et al. 2008). In our previous study, we reported that PspR (referred to as the PPS-Like protein at that time) potentially acts as a negative regulator of the *phaC1* gene by binding to its promoter region (Chen et al. 2020). In this study, we provide evidence for the negative regulation of PspR on the promoter region of *phaRP* and suggest a possibility of functional conversion (from PPS or RPH to PspR) according to the structure modeling and activity detection.

RPH has a great similarity with PPS and PPDK in the His domain (Stogios et al. 2016), revealing that they may have evolved from a common ancestor. RPH is capable of inactivating rifamycin by transferring the phosphate group from ATP to rifamycin (Fig. 1a). According to our predicted model, PspR has both the similar His domain and the ATP-binding domain with RPH, which attests to the closer relationship between PspR and RPH. We expressed RPH_*Lm*_ protein (Fig. S2) and set RPH_*Lm*_ as the positive control to detect the potential phosphate transferase activity of PspR. However, as shown in Fig. S3b, no RIF-P was generated in the reaction involving PspR, indicating PspR lacks rifampicin phosphotransferase activity. According to the differences in the substrate-binding domains between PspR and RPH_*Lm*_, we speculate that the LD region of the PspR may have undergone mutations and cannot bind to rifampicin. Instead, it has acquired the function of DNA binding.

Interestingly, PspR might have converted from a member of the PEP utilization enzyme into a regulatory protein, which can bind to the P_*phaR*_ region to inhibit the expression of *phaR* and *phaP*, and then affect the synthesis of PHA. Therefore, PhaR plays an important role in the regulatory network of PspR for PHA biosynthesis. In our previous study, PhaR has been proven to be a bifunctional protein that plays the central role in the regulation of PHA accumulation and granule formation in *H. mediterranei*, and homologs of PhaR are widespread in haloarchaea (Cai et al. 2015). According to the ubiquity of PhaC protein in PHA-synthesizing strains (Lu et al. 2008; Williams et al. 2017; Zhao et al. 2015), we analyzed the co-distribution of PspR, PhaR and PhaC protein in halophilic archaea based on the unrooted tree of PspR (Chen et al. 2019). Among the 13 haloarchaea strains containing PspRs (Table 1), 10 strains also harbored the PhaC protein besides *H. mediterranei*. Additionally, at least 3 strains of them have been reported to synthesize PHA: *Natronococcus occultus*, *Halorubrum lacusprofundi*, and *Halogeometricum borinquense* (Legat et al. 2010; Salgaonkar and Bragança 2015; Williams et al. 2017). Furthermore, for *Natronococcus amylolyticus*, *Halogranum rubrum*, *Halalkalicoccus jeotgali*, *Halalkalicoccus paucihalophilus*, and *Natrinema salaciae*, the same genus of these strains, were identified as capable of synthesizing PHA. Among them, PhaR homologs from these strains show significant similarity with identities over than 50%, containing conserved AbrB-like domain (Fig. S4). Only one species of them, *Halorubrum lacusprofundi*, has lower identities with 23.4%. Taken together, the regulation of PHA synthesis by PspR-PhaR may be widespread in PHA-accumulating haloarchaea.

**Table 1:** Distribution of PhaC and PhaR in haloarchaea with PspR

Haloarchaea expressing PspR	PhaC^a^	PhaR^b^	PHA^c^	Reference
*Natronococcus amylolyticus*	Y	Y	y	(Legat et al. 2010)
*Natronolimnobius innermongolicus*	Y	Y	NR	
*Natronococcus occultus*	Y	Y	Y	(Legat et al. 2010)
*Haladaptatus* sp*.*	Y	Y	NR	
*Halorubrum lacusprofundi*	Y	y	Y	(Williams et al. 2017)
*Halogranum rubrum*	Y	Y	y	(Zhao et al. 2015)
*Halogeometricum borinquense*	Y	Y	Y	(Salgaonkar and Bragança 2015)
*Halalkalicoccus jeotgali*	Y	Y	y	(Han et al. 2010a, b)
*Halalkalicoccus paucihalophilus*	Y	Y	y	(Han et al. 2010a, b)
*Natrinema salaciae*	Y	Y	y	(Karray et al. 2021)

^a^Y means this strain expresses PhaC

^b^Y means this strain expresses PhaR homolog with identities over 50%. y means this strain expresses PhaR homolog with identities less than 50%

^c^Y means that this strain can accumulate PHA according to the reference. y means that there are some strains of the same genus, which can accumulate PHA. NR means that there are no reports about this

With the increasing demand for eco-friendly alternatives to petroleum-based plastics, genetic manipulation of haloarchaea to construct high-yielding PHA strains has become a promising approach to meet the global market demand for PHA (Ye et al. 2018). However, the available models for the genetic manipulation of haloarchaea are still limited (Mitra et al. 2020), posing a significant challenge to PHA production. In this context, our findings on the regulatory role of PspR in PHA biosynthesis and its co-distribution with the PhaC protein in PHA-synthesizing haloarchaea, shed new light on the potential use of PspR as a novel regulatory element for genetic manipulation of these microorganisms. Furthermore, our research expands the current understanding of PHA biosynthesis and regulation in haloarchaea, which may aid in the development of more efficient and sustainable PHA production processes.

## Supplementary Information

Below is the link to the electronic supplementary material.

Supplementary file1 (PDF 1875 KB)

## Data Availability

All data accompanying this research are presented directly in the manuscript and supplementary material.
