# Supplementary material for: Unveiling the repressive mechanism of a PPS-like regulator (PspR) in polyhydroxyalkanoates biosynthesis network
Source: Appl Microbiol Biotechnol. 2024 Mar 18;108(1):265. doi: 10.1007/s00253-024-13100-x (PMC10948481; doi:10.1007/s00253-024-13100-x)
Supplement: Supplementary file 1 — Supplementary file1 (PDF 1875 KB) [file 253_2024_13100_MOESM1_ESM.pdf]

Supplementary Information

Unveiling the Repressive Mechanism of a PPS-like Regulator (PspR) in Polyhydroxyalkanoates Biosynthesis Network

Junyu Chen[1], Yinglu Cui[1,2], Shengjie Zhang[1], Bian Wu[1,2], Jing Han[1,2*], Hua Xiang[1,2*]

[1] State Key Laboratory of Microbial Resources, Institute of Microbiology, Chinese Academy of Sciences, Beijing, 100101, China

[2] College of Life Science, University of Chinese Academy of Sciences, 100049, Beijing, People's Republic of China

[*]Correspondence: hanjing@im.ac.cn (for J. Han) or xiangh@im.ac.cn (for H. Xiang)

Table S1 Strains and plasmids used in this study.

Strains	Relevant characteristics	Source or reference
Escherichia coli JM109	*recA1 supE44 endA1 hsdR17 gyrA96 relA1 thi*	(Sambrook et al. 1989)
E. coli JM110	*dam dcm* mutant of *E. coli* JM109	Novagen
E. coli BL21 (DE3)	F^-ompT hsdS($r_B^- m_B^-$) *gal dcm*(DE3)	Novagen
H. mediterranei	*H. mediterranei* ATCC 33500	(Lu et al. 2008)
ΔEPS	EPS synthesis gene cluster deletion and *pyrF*-deleted mutant of wild-type strain *H. mediterranei* ATCC 33500	(Zhao et al. 2013)
ΔEPSΔ*pspR*	*pspR* deletion mutant of ΔEPS	(Chen et al. 2019)
ΔEPSΔ*pspR*(PspR-Myc)	overexpression of PspR with Myc-tag in ΔEPSΔ*pspR* strain	This study
ΔEPSΔRPEC::*gfp*	PHA related genes cluster ORF replaced by GFP ORF *in situ* in ΔEPS strain	This study
ΔEPSΔ*pspR*ΔRPEC::*gfp*	PHA related genes cluster ORF replaced by GFP ORF *in situ* in ΔEPSΔ*pspR* strain	This study
ΔEPSΔ*pspR*ΔRPEC::*gfp*(502)	ΔEPSΔ*pspR*ΔRPEC::*gfp* strain with pWL502 plasmid	This study
ΔEPSΔ*pspR*ΔRPEC::*gfp*(PspR)	overexpression of PspR in ΔEPSΔ*pspR*ΔRPEC::*gfp* strain	This study
ΔEPSΔ*pspR*ΔRPEC::*gfp*(E_{377}A-R_{519}A)	overexpression of mPspR (E_{377}A-R_{519}A) in ΔEPSΔ*pspR*ΔRPEC::*gfp* strain	This study
ΔEPSΔ*pspR*ΔRPEC::*gfp*(W_{360}A-W_{380}A)	overexpression of mPspR (E_{377}A-R_{519}A) in ΔEPSΔ*pspR*ΔRPEC::*gfp* strain	This study
Haloferax volcanii H1424	*ΔpyrE2 ΔhdrB pitANph Δmrr cdc48d-Ct*	(Stroud et al. 2012)

Plasmids	Relevant characteristics	Source or reference
pHFX	4.0 kb; integration vector containing *pyrF* and its native promoter, Amp^r	(Liu et al. 2011)
pHFX-GFP	5.2 kb; integration vector of pHFX for replacement of *gfp*	This study
pWL502	7.9 kb; shuttle vector with *pyrF* marker, Amp^r	(Liu et al. 2011)
pWL502-PspR	10.8 kb; overexpression of PspR	This study

pWL502-E_{377}A-R_{519}A	10.8 kb; overexpression of mPspR	This study
pWL502-W_{360}A-W_{380}A	10.8 kb; overexpression of mPspR	This study
pWL502-PspR-Myc	10.6 kb; overexpression of PspR by with Myc-tag	This study
pTA06	8.0 kb; expression vector with N-terminal His_6 tag and promoter P_{phaR}	(Liu et al. 2015)
pTA06-PspR	10.6 kb; expression plasmid of pTA06 containing *pspR*	This study
pTA06-F_{340}A	10.6 kb; expression plasmid of pTA06 containing mutant *pspR*	This study
pTA06-W_{360}A	10.6 kb; expression plasmid of pTA06 containing mutant *pspR*	This study
pTA06-W_{380}A	10.6 kb; expression plasmid of pTA06 containing mutant *pspR*	This study
pTA06-E_{377}A-R_{519}A	10.6 kb; expression plasmid of pTA06 containing mutant *pspR*	This study
pTA06-W_{360}A-W_{380}A	10.6 kb; expression plasmid of pTA06 containing mutant *pspR*	This study
pTA06-F_{340}A-W_{360}A-W_{380}A	10.6 kb; expression plasmid of pTA06 containing mutant *pspR*	This study

Table S2. Primers used in this study.

Primers	Sequence (5'-3')	Description
RPEC-NF	GCGTGGCGTGGATGAGATATCGAGCTCCTTCTGCGAACGTTTCGTC	Amplification of upstream and downstream flanking regions of *phaR-phaP-phaEC* genes with GFP inserted into pHFX to construct pHFX-GFP
RPEC-GFP-NR	GTTCTTCTCCTTTACTCATCTCCTAACTCGGTGTTGTACC	
RPEC-GFP-CF	GGATGAACTATACAAATAATCGTTTTTTCGACGTGAAAATCG	
RPEC-CR	TATAGGGAGAAGCTTGCATGCCCCGGCCTAATCGATAGC	
RPEC-GFP-F	CGAGTTAGGAGATGAGTAAAGGAGAAGAAC	
RPEC-GFP-R	CGTCGAAAAAACGATTATTTGTATAGTTCATCC	
ChIP-PspR-ORF-F	GACAACAACCCCCCATGGATCTGCTGATGCAATGTGGCTCACA	Amplification of *pspR* with Myc-tag inserted into

ChIP-PspR-ORF-Myc-R	GCAAGGGAACCGCACACAAGAAAACTCACAGATCCTCCTCGCTGATGAGCTTCTGCTCGTACCGTTCGGACCGGTCGAG [a]	pWL502 to construct pWL502-PspR-Myc
Myc-F	GAGCAGAAGCTCATCAGCGAGGAGGATCTGTGA	
Myc-R	TCACAGATCCTCCTCGCTGATGAGCTTCTGCTC	
ChIP-Myc-F	CGGTCCGAACGGTACGAGCAGAAGCTCATCAGC	
ChIP-Myc-R	CGCACACAAGAAAACGGTACCTCACAGATCCTCCTCGCTGAT	
phaR-Pro-F	GGTCGTTACTGCGGCTCT	qPCR analysis of P_{phaR} region fragment
phaR-Pro-R	CCACCTCGCATCGTTTGA	
phaC1-Pro-F	AGGTTCCACATCGTAATCTCG	qPCR analysis of P_{phaC1} region fragment
phaC1-Pro-R	GTAAACGGGTTCATGGTCAT	
gvpA -Pro-F	TGAATCGGGCTGAACCAT	qPCR analysis of P_{gvpA} region fragment
gvpA-Pro-R	TAGGCAGGGTTTGGTGGG	
kch-Pro-F	CGGAACCAGCCACTGAAC	qPCR analysis of P_{kch} region fragment
kch-Pro-R	CGCGTGGCTACCCATATT	
pspR-F	CGCCATATGAACGAGAGGGGAGACACGCCAG	Amplification of *pspR* inserted into pTA06 to construct pTA06-PspR
pspR-R	CGCGGATCCGTACCGTTCGGACCGGTCGAGG	
pspR-360WAF	CTCGTCGTAGACTGGGCGCGGGAACTGCTCAAT [b]	Amplification of mutant *pspR* inserted into pTA06 to construct pTA06-F_{340}A and pTA06-F_{360}A
pspR-360WAR	ATTGAGCAGTTCCCGCGCCCAGTCTACGACGAG	
pspR-WA380F	AACACCGAAGCACCGGCGGCGGTCGAAGCCGGC	Amplification of mutant *pspR* inserted into pTA06 to construct pTA06-W_{380}A
pspR-WA380R	GCCGGCTTCGACCGCCGCCGGTGCTTCGGTGTT	
pspR-EA377F	CGTTCAGGGAACACCGCGGCACCGTGGGCGGTC	Amplification of mutant *pspR* inserted into pTA06 to construct pTA06-E_{377}A
pspR-EA377R	GACCGCCCACGGTGCCGCGGT	

	GTTCCCTGAACG	
pspR-RA519F	TTGACAGTACTCAGTGCGTTAC TCCCCCGTATC	Amplification of mutant *pspR* inserted into pTA06 to construct pTA06-R_{519}A
pspR-RA519R	GATACGGGGGAGTAACGCACT GAGTACTGTCAA	

[a] Underlines stands for Myc sequence.

[b] Double underlines stand for protein mutant sites.

Table S3 DNA enrichment assay of the PspR-Myc fusion protein.

Ct	PphaR			PphaC1			PgvpA			Pkch			7S		
**output**	26.51	26.93	26.97	26.83	26.92	26.99	26.35	26.78	27.29	25.44	25.45	26.92	25.82	25.97	26.31
**mock**	27.83	27.83	28.69	26.73	27.07	28.23	26.45	26.49	26.5	25.58	25.82	26.06	25.93	25.94	26.22
**input**	11.1	11.16	11.32	11.25	11.3	11.38	11.24	11.41	11.49	11.17	11.28	12.33	10.99	11.02	11.03

	PphaR			PphaC1			PgvpA			Pkch			7S		
**$\Delta Ct_{output}=Ct_{output}-Ct_{input}$**	15.41	15.77	15.65	15.58	15.62	15.61	15.11	15.37	15.8	14.27	14.17	14.59	14.83	14.95	15.28
**$\Delta Ct_{mock}=Ct_{mock}-Ct_{input}$**	16.73	16.67	17.37	15.48	15.77	16.85	15.21	15.08	15.01	14.41	14.54	13.73	14.94	14.92	15.19
**$\Delta\Delta Ct=\Delta Ct_{output}-\Delta Ct_{mock}$**	-1.32	-0.9	-1.72	0.1	-0.15	-1.24	-0.1	0.29	0.79	-0.14	-0.37	0.86	-0.11	0.03	0.09
**$-\Delta\Delta Ct$**	1.32	0.9	1.72	-0.1	0.15	1.24	0.1	-0.29	-0.79	0.14	0.37	-0.86	0.11	-0.03	-0.09
**$2^{(-\Delta\Delta Ct)}$**	2.496661	1.866066	3.294364	0.933033	1.109569	2.361985	1.071773	0.817902	0.578344	1.101905	1.292353	0.550953	1.079228	0.97942	0.939523
**mean, SD**	2.552364	0.715776		1.468196	0.779061		0.822673	0.246749		0.981737	0.385031		0.99939	0.071962	

Table S4 GFP expression is upregulated in ΔEPSΔ*pspR* ΔRPEC::*gfp* strain

	Exponential phase						Stationary phase					
**Strain**	ΔEPSΔRPEC::*gfp*			ΔEPSΔ*pspR*ΔRPEC::g*fp*			ΔEPSΔRPEC::*gfp*			ΔEPSΔ*pspR*ΔRPEC::g*fp*		
**OD600**	0.276	0.266	0.271	0.288	0.283	0.294	1.882	1.856	1.853	2.007	2.009	2.018
**488,509**	1142	1152	1145	3814	3891	3920	18839	19036	18028	22560	22157	22311
**RFU**	4137.681	4330.827	4225.092	13243.0556	13749.117	13333.333	10010.1	10256.47	9729.088	11240.6577	11028.87008	11055.996
**Mean, SD**	4231.2	96.71771		13441.8352	269.91468		9998.55	263.8783		11108.50794	115.2459254	
**Fold change**				3.17683746						1.111011923		

Table S5 Calculation of DNA-binding efficiency of mPspR to P_{phaR}.

Mutation site	Fig. 5a	Fig. 5b	Fig. 5c	Average[a]
PspR (wt[b])	1	1	1	1
$E_{377}A-R_{519}A$[c]	0.63	0.47	0.45	0.52 ± 0.09***
$W_{340}A$	0.75	0.45	0.69	0.63 ± 0.16**
$W_{360}A$	1.85	1.02	1.55	1.47 ± 0.17
$W_{380}A$	1.96	1.11	1.4	1.49 ± 0.19
$W_{360}A-W_{380}A$	1.75	1.77	1.65	1.72 ± 0.06**
$W_{340}A-W_{360}A-W_{380}A$	1.63	1.76	1.47	1.62 ± 0.15***

[a]All data are expressed as means ± standard deviations of independent repeated experiments results. Statistical significance is defined as ***P < 0.001, and **P <0.01.

[b]wt is the wild type of PspR.

[c]Number is the mutation site of PspR.

Table S6 Different binding specificity of mPspRs to P_{phaR} region determined by fluorescent reporter system.

Strain	ΔEPSΔpspRΔRPEC::gfp(502)			ΔEPSΔpspRΔRPEC::gfp(PspR)			ΔEPSΔpspRΔRPEC::gfp(E377A-R519A)			ΔEPSΔpspRΔRPEC::gfp(E377A-R519A)		
**OD600**	0.257	0.264	0.272	0.276	0.268	0.272	0.264	0.277	0.271	0.269	0.262	0.261
**488,509**	3808	3938	3793	1155	1212	1052	2188	2112	2128	905	787	898
**RFU**	14817.1206	14916.6667	13944.85294	4184.782609	4522.38806	3867.647059	8287.878788	7624.548736	7852.398524	3364.312268	3003.816794	3440.613027
**Mean, SD**	14559.5467	534.662236		4191.605909	327.4238272		7921.608683	337.037422		3269.580696	233.2987147	
**Fold change**				**contrast=1**			1.889874395			0.780030558		

Figure

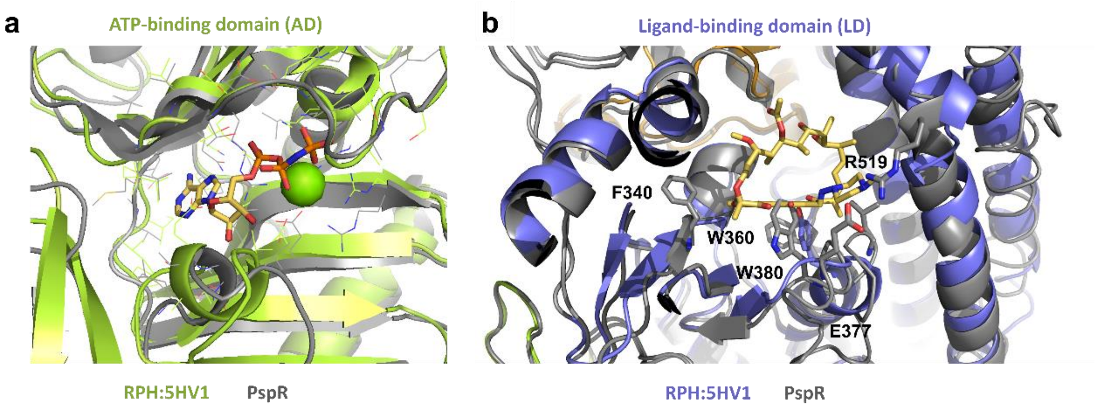

Fig. S1 Structure superimposition of predicted PspR model with RPH$_{Lm}$-rifampin bound complex (PDB ID: 5HV1). (a) Structure superimposition of ATP-binding domain (AD) in PspR model with RPH-ATP bound complex. PspR model and RPH-rifampin complex are colored grey and green, respectively. ATP is shown as stick; Mg^{2+} is shown as a green sphere. (b) Structure superimposition of Ligand-binding domain (LD) in PspR model with RPH-rifampin bound complex. PspR model and RPH-rifampin complex are colored grey and hyacinth, respectively. Rifampin is shown as stick. Candidate key residues in LD region are shown, W: tryptophan, R: arginine, E: glutamic acid.

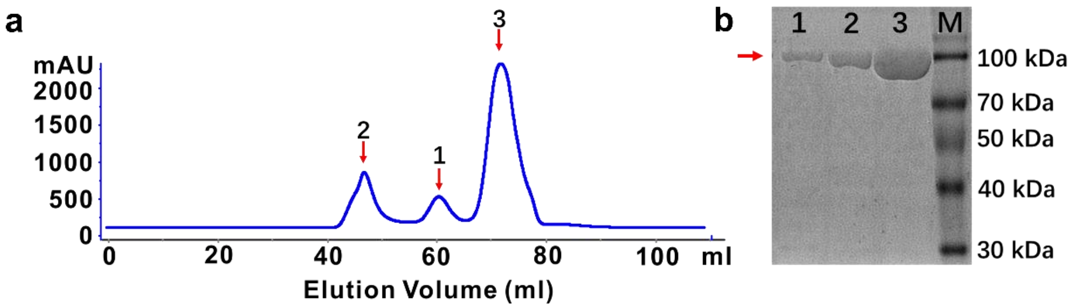

Fig. S2 Purification of RPH$_{Lm}$ expressed in by MS (a) and SDS-PAGE result (b). Both in (A) and (B), lane 3 is RPH protein monomer; lane 1 and 2 are different polymerization states.

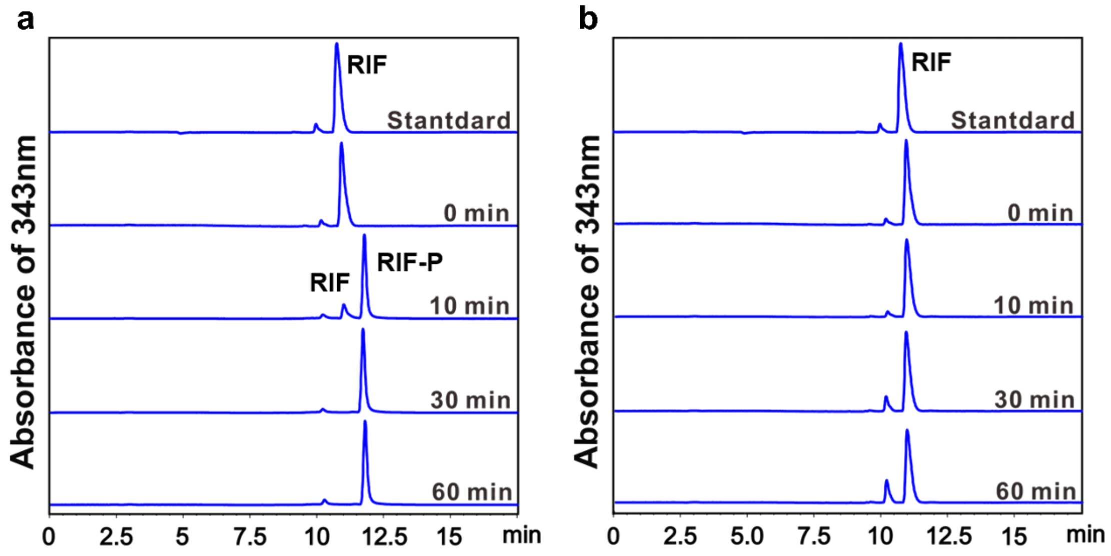

Fig. S3 Detection of phosphotransferase activity of RPH*Lm* (a) and PspR (b) by HPLC. Rifampin (RIF) is the substrate, and phosphorylated rifampin (RIF-P) is the product. HPLC was used to detect the substrate (RIF) and product (RIF-P) during the reaction at 0 min, 10 min, 30 min and 60 min time points. The retention time of RIF is about 11 min, and the retention time of RIF-P is about 12 min.

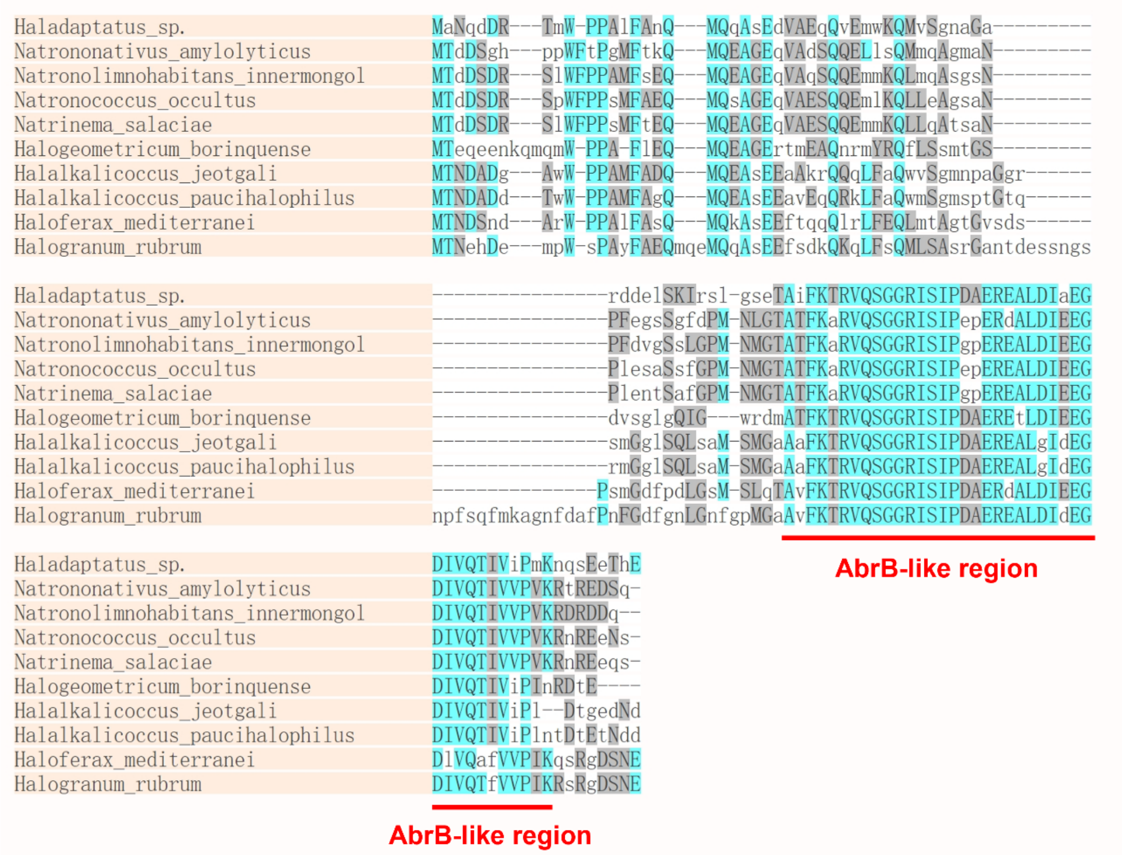

Fig. S4 Multiple alignments of amino acid sequences of PhaR homologs from nine representative haloarchaea species: *Haloferax mediterranei*, *Natrononativus amylolyticus*, *Natronolimnobius innermongolicus*, *Natronococcus occultus*, *Haladaptatus* sp., *Halogranum rubrum*, *Halogeometricum borinquense*, *Halalkalicoccus jeotgali*, *Halalkalicoccus paucihalophilus* and *Natrinema salaciae*. The AbrB-like region is indicated by red bar. Sequence analysis and database. Query sequences were accessed from the National Center for Biotechnology Information (NCBI) Protein Database. Sequence homology was analyzed *via* the Uniprot BLAST server (https://www.uniprot.org/). The sequences of each PhaR homolog are listed as follows:

>WP_004056141.1 *Haloferax mediterranei*

MTNDSNDARWPPALFASQMQKASEEFTQQQLRLFEQLMTAGTGVSDSPSMG
DFPDLGSMSLQTAVFKTRVQSGGRISIPDAERDALDIEEGDLVQAFVVPIKQSR
GDSNE

>WP_255167741.1 *Natrononativus amylolyticus*

MTDDSGHPPWFTPGMFTKQMQEAGEQVADSQQELLSQMMQAGMANPFEGS
SGFDPMNLGTATFKARVQSGGRISIPEPERDALDIEEGDIVQTIVVPVKRTREDS
Q

>WP_007261346.1 *Natronolimnohabitans innermongolicus*

MTDDSDRSLWFPPAMFSEQMQEAGEQVAQSQQEMMKQLMQASGSNPFDVG
SSLGPMNMGTATFKARVQSGGRISIPGPEREALDIEEGDIVQTIVVPVKRDRDD
Q

>WP_015320638.1 *Natronococcus occultus*

MTDDSDRSPWFPPSMFAEQMQSAGEQVAESQQEMLKQLLEAGSANPLESASS
FGPMNMGTATFKARVQSGGRISIPEPEREALDIEEGDIVQTIVVPVKRNREENS

>WP_066146951.1 *Haladaptatus* sp.

MANQDDRTMWPPALFANQMQQASEDVAEQQVEMWKQMVSGNAGARDDEL
SKIRSLGSETAIFKTRVQSGGRISIPDAEREALDIAEGDIVQTIVIPMKNQSEETH
E

>WP_241211152.1 *Halorubrum lacusprofundi*

MNTKNGLRNSKKRLKQRKTNSTNIAVILITYYFISPSRDMPRITTKGQVTIPKEI
RETLGIEPGDEIAFEEVSSGYKIQKKEPTTADGNDPFAKYRGSAESDETMPER
MRRLRREYPRDVGDDCDDESEAEA

>WP_089865813.1 *Halogranum rubrum*

MTNEHDEMPWSPAYFAEQMQEMQQASEEFSDKQKQLFSQMLSASRGANTDE
SSNGSNPFSQFMKAGNFDAFPNFGDFGNLGNFGPMGAAVFKTRVQSGGRISIP
DAEREALDIDEGDIVQTFVVPIKRSRGDSNE

>WP_006056100.1 *Halogeometricum borinquense*

MTEQEENKQMQMWPPAFLEQMQEAGERTMEAQNRMYRQFLSSMTGSDVSG
LGQIGWRDMATFKTRVQSGGRISIPDAERETLDIEEGDIVQTIVIPINRDTE

>WP_008417219.1 *Halalkalicoccus jeotgali*

MTNDADGAWWPPAMFADQMQEASEEAAKRQQQLFAQWVSGMNPAGGRSM
GGLSQLSAMSMGAAAFKTRVQSGGRISIPDAEREALGIDEGDIVQTIVIPLDTG
EDND

>WP_066382202.1 *Halalkalicoccus paucihalophilus*

MTNDADDTWWPPAMFAGQMQEASEEAVEQQRKLFAQWMSGMSPTGTQRM

GGLSQLSAMSMGAAAFKTRVQSGGRISIPDAEREALGIDEGDIVQTIVIPLNTD

TETNDD

>WP_090612128.1 *Natrinema salaciae*

MTDDSDRSLWFPPSMFTEQMQEAGEQVAESQQEMMKQLLQATSANPLENTS

AFGPMNMGTATFKARVQSGGRISIPGPEREALDIEEGDIVQTIVVPVKRNREEQ

S

Supplementary Reference

Chen JY, Mitra R, Zhang SJ, Zuo ZQ, Lin L, Zhao DH, Xiang H, Han J (2019) Unusual phosphoenolpyruvate (PEP) synthetase-like protein crucial to enhancement of polyhydroxyalkanoate accumulation in *Haloferax mediterranei* revealed by dissection of PEP-pyruvate interconversion mechanism. Appl Environ Microbiol 85(19) https://doi.org/10.1128/AEM.00984-19

Liu GM, Hou J, Cai SF, Zhao DH, Cai L, Han J, Zhou J, Xiang H (2015) A patatin-like protein associated with the polyhydroxyalkanoate (PHA) granules of *Haloferax mediterranei* acts as an efficient depolymerase in the degradation of native PHA. Appl Environ Microbiol 81(9):3029-38 https://doi.org/10.1128/aem.04269-14

Liu HL, Han J, Liu XQ, Zhou J, Xiang H (2011) Development of *pyrF*-based gene knockout systems for genome-wide manipulation of the archaea *Haloferax mediterranei* and *Haloarcula hispanica*. J Genet Genomics 38(6):261-9 https://doi.org/10.1016/j.jgg.2011.05.003

Lu QH, Han J, Zhou LG, Zhou J, Xiang H (2008) Genetic and biochemical characterization of the poly(3-hydroxybutyrate-*co*-3-hydroxyvalerate) synthase in *Haloferax mediterranei*. J Bacteriol 190(12):4173-80 https://doi.org/10.1128/JB.00134-08

Sambrook J, Fritsch EF, Maniatis T (1989) Molecular Cloning: A laboratory manual, 2nd edn. Cold Spring Harbor Laboratory Press, Cold Spring Harbor, N.Y

Stroud A, Liddell S, Allers T (2012) Genetic and biochemical identification of a novel single-stranded DNA-binding complex in *Haloferax volcanii*. Front Microbiol

3(224) https://doi.org/10.3389/fmicb.2012.00224

Zhao DH, Cai L, Wu JH, Li M, Liu HL, Han J, Zhou J, Xiang H (2013) Improving polyhydroxyalkanoate production by knocking out the genes involved in exopolysaccharide biosynthesis in *Haloferax mediterranei*. Appl Microbiol Biotechnol 97(7):3027-36 https://doi.org/10.1007/s00253-012-4415-3

3
